# Kounis Syndrome Secondary to Medicine-Induced Hypersensitivity

**DOI:** 10.1155/2021/4485754

**Published:** 2021-10-01

**Authors:** Parackrama Karunathilake, Udaya Ralapanawa, Thilak Jayalath, Shamali Abeyagunawardena

**Affiliations:** Department of Medicine, Faculty of Medicine, University of Peradeniya, Kandy, Sri Lanka

## Abstract

**Introduction:**

Kounis syndrome is the concurrence of an acute coronary syndrome (ACS) caused by coronary vasospasms, acute myocardial infarctions, or stent thromboses in case of allergic or hypersensitivity reactions. Kounis syndrome is mediated by mast cells that interact with macrophages and T-lymphocytes, causing degranulation and inflammation with cytokine release. It is a life-threatening condition that has many trigger factors and is most commonly caused by medicines. *Case Presentation*. A 71-year-old male was admitted with a fever of five days' duration associated with cellulitis, for which he had been treated with clindamycin and flucloxacillin before admission. He was a diagnosed patient with hypertension and dyslipidemia five years ago. After taking the antibiotics, he had developed generalized itching followed by urticaria suggesting an allergic reaction. Therefore, he was admitted to the hospital. After admission, he developed an ischaemic-type chest pain associated with autonomic symptoms and shortness of breath. An immediate ECG was taken that showed ST-segment depressions in the chest leads V4–V6, confirmed by a repeat ECG. Troponin I was 8 ng/mL. Acute management of ACS was started, and prednisolone 10 mg daily dose was given. After complete recovery, the patient was discharged with aspirin, clopidogrel, atorvastatin, metoprolol, losartan, isosorbide mononitrate, and nicorandil. Prednisolone 10 mg daily dose was given for five days after discharge.

**Conclusion:**

In immediate hypersensitivity, with persistent cardiovascular instability, Kounis syndrome should be considered, and an electrocardiogram and other appropriate assessments and treatments should be initiated. Prompt management of the allergic reaction and the ACS is vital for a better outcome of Kounis syndrome.

## 1. Introduction

Cardiovascular manifestations associated with allergy, hypersensitivity, and anaphylactic or anaphylactoid reactions began to emerge seven decades ago in the medical literature. However, the clinical condition of allergic angina syndrome, which progresses to allergic acute myocardial infarction, was not described until 1991 [1]. Allergic angina syndrome and allergic myocardial infarction are ubiquitous diseases that affect patients of any age. They cover a broad spectrum of mast cell-activation disorders that are referred to as Kounis syndrome. The definition of this syndrome is the concurrence of an acute coronary syndrome (ACS) in the setting of allergic or hypersensitivity and anaphylactic or anaphylactoid insults. These cases of ACS may occur due to coronary spasms, acute myocardial infarctions, or stent thromboses [2]. The primary inflammatory cells involved in the development of Kounis syndrome are mast cells that interact with macrophages and T-lymphocytes. It causes mast cell degranulation and release of inflammatory mediators such as histamine, neutral proteases chymase, tryptase, heparin, and cathepsin-D, with increased production of leukotrienes [3].

Medicines represent the most common trigger for Kounis syndrome in clinical practice. The most commonly reported medicines include nonsteroidal anti-inflammatory drugs (e.g., ibuprofen), antibiotics such as amoxicillin/clavulanate, and anticancer drugs (e.g., fluorouracil) [4]. Kounis syndrome is a complex situation involving two severe circumstances, an acute allergic reaction and an acute coronary event. Thus, the prognosis of Kounis syndrome is relatively poor [5]. Therefore, the management should address both prior given concerns that envisage the complexity of the management. Herein, we report a case of Kounis syndrome possibly triggered by antibiotics administered due to cellulitis of the right lower limb.

## 2. Case Presentation

**Figure 1:**
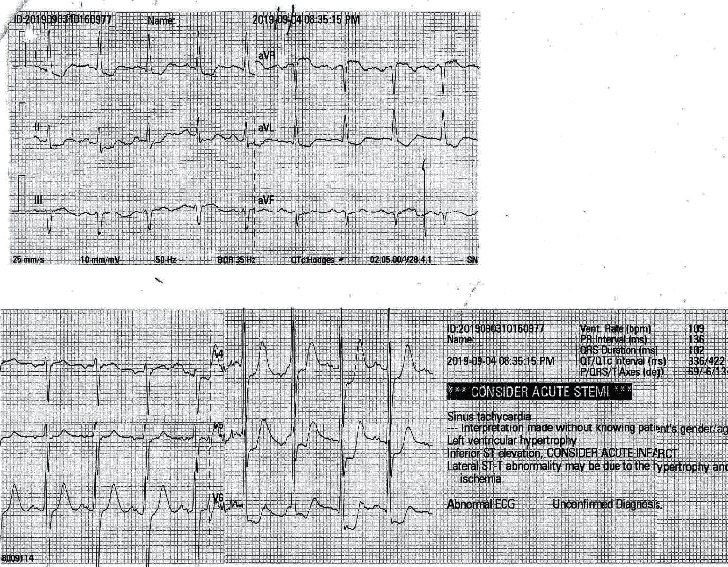
Electrocardiogram taken immediately after admission, showing ST-segment depression in the chest leads, V4–V6.

**Figure 2:**
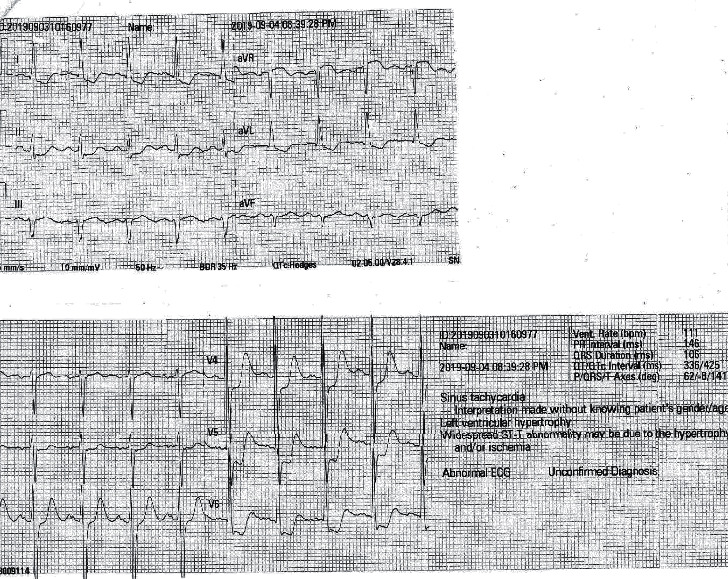
Repeat electrocardiogram taken after admission, showing ST-segment depression in the chest leads, V4–V6.

A 71-year-old male was admitted with a fever of five days' duration and a reddish swollen right lower limb that had occurred following a wound caused by a leech bite. He had initially consulted a general practitioner before admission who had diagnosed cellulitis, and the patient had been prescribed clindamycin and flucloxacillin on day 2 of the fever. After taking the antibiotics, he had developed generalized itching followed by urticaria. However, there had not been any shortness of breath. Then, antibiotics had been changed on day 4 of fever by the general practitioner due to the possibility of an allergic reaction. Then, he was given cephalexin. However, the allergic symptoms were persisting, and the wound condition had not been improved. Therefore, he was admitted to the local hospital on day 5 of fever. After being admitted to the hospital, he developed ischaemic-type chest pain associated with autonomic symptoms and shortness of breath. The pain was slightly reduced by a glyceryl trinitrate sublingual tablet given. An electrocardiogram (ECG) was taken immediately, showing ST-segment depressions in the chest leads, V4–V6 (Figure 1). A repeat ECG confirmed similar findings (Figure 2).

**Figure 3:**
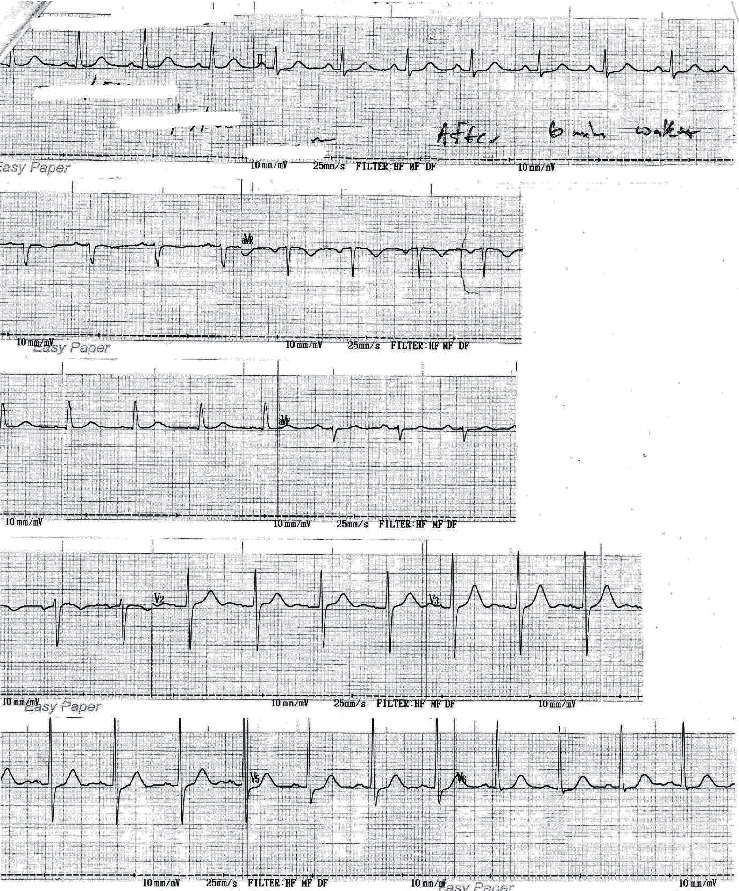
Electrocardiogram taken at the tertiary-care center after treatment showing normal findings.

The acute management of ACS was carried out at the local hospital, and the patient was transferred to a tertiary-care center for further management. At the tertiary-care center, the antiplatelets and statins were continued. Enoxaparin 60 mg, six doses, and prednisolone 10 mg daily dose were given. ECGs (Figure 3) taken at the tertiary-care center showed normal findings. Troponin I level was initially 8 ng/mL and then reduced to 0.28 ng/mL four days after the onset of chest pain.

He was a patient diagnosed with hypertension and dyslipidemia for five years without any hypertensive complications. He had also taken treatment for gastro-oesophageal reflux disease for seven years. Nevertheless, he did not have any previous history of diabetes mellitus, transient ischaemic attacks, or cerebrovascular accidents. He had a significant history of allergies to tetracycline, amoxicillin, flucloxacillin, clindamycin, and mushrooms. He did not have any family history of ischaemic heart disease. He was a heavy smoker who had consumed 40 pack-years of cigarettes and was an alcoholic who had taken four to five bottles of toddy per day for thirty years which amounted to 15 units per day; however, he claimed abstinence for five years.

After full recovery, he was discharged with aspirin, clopidogrel, atorvastatin, metoprolol, losartan, isosorbide mononitrate, and nicorandil. Prednisolone 10 mg daily dose was prescribed for five days.

He was next followed up at the cardiac rehabilitation clinic. Six months after the initial presentation, he presented again with stable angina, and the exercise ECG showed stage II inferolateral ischemia. A 2D echocardiogram revealed an ejection fraction of 59% with normal left ventricular systolic dysfunction. We calculated his TIMI score, which was 6 out of 7. Although a coronary angiogram was planned, the patient defaulted follow-up.

## 3. Discussion

Kounis syndrome was defined in 1991 as “the coincidental occurrence of chest pain and allergic reactions accompanied by clinical and laboratory findings of classic angina pectoris caused by inflammatory mediators released during the allergic insult” [6]. It comprises the whole clinical spectrum of acute myocardial ischemia, from stable angina to acute myocardial infarction, which coincides with “allergic” (hypersensitivity, anaphylactic, or anaphylactoid) reaction [6].

Three variants of Kounis syndrome have been described. The type I variant includes normal or nearly normal coronary arteries without risk factors for coronary artery disease. The type II variant includes the culprit but quiescent preexisting atheromatous disease. The type III variant includes coronary artery stent thrombosis in which aspirated thrombus specimens are stained with hematoxylin-eosin and Giemsa that demonstrate the presence of eosinophils and mast cells [2]. Type III variant has two subtypes; subtype *a* includes patients with coronary stent thrombosis, and subtype *b* includes patients with stent restenosis due to allergic inflammation [7]. Recently, a new variant IV of Kounis syndrome has been proposed, not scientifically established yet [8].

Involvement of the cardiovascular system in anaphylaxis occurs in two-thirds of cases [9]. Nevertheless, the incidence of Kounis syndrome during acute allergic reactions has not been precisely established [5]. In a prospective study performed at an emergency department, 27 out of 793 patients consulting for allergic reactions were diagnosed with Kounis syndrome, rendering an incidence of 3.4% [10]. Myocardial injury detected by elevation of troponin I was diagnosed in 22 cases (7.3%) in another retrospective study reviewing 300 cases of anaphylaxis [11].

Although anaphylactic reactions associated with cardiovascular alterations are frequent and transient, in some cases, they may result in extensive and life-threatening myocardial damage. In such incidence, many mast cells have been found in the tunica media and the adventitia of large coronary vessels and small intramural coronary arteries. During anaphylaxis, coronary hypoperfusion caused by systemic vasodilation, plasma leakage, volume loss due to increased vascular permeability, and reduced venous return can contribute to cardiac output suppression, leading to further myocardial damage and ventricular dysfunction [12].

The primary inflammatory cells involved in the development of Kounis syndrome are mast cells that interact with macrophages and T-lymphocytes via multidirectional stimuli. During allergy, hypersensitivity, and anaphylaxis, mast cell degranulation occurs, and various stored and newly formed inflammatory mediators are released locally and to the systemic circulation. These inflammatory mediators include biogenic amines such as histamine, chemokines, enzymes such as the neutral proteases chymase and tryptase, cathepsin-D, peptides, proteoglycans, cytokines, growth factors, and arachidonic acid products such as leukotrienes, thromboxane, prostacyclin, PAF, and tumor necrosis factor-*α* (TNF-*α*) [1]. It is shown that mast cells in human coronary atheromas contain TNF-*α* [13]. Mast cells can be typically activated via several pathways, which may contribute to Kounis syndrome. One is through allergen cross linking with allergen-specific IgE bound to high-affinity Fc epsilon receptor. Non-IgE-mediated mast cell degranulation can also occur via the anaphylatoxins, including complement C1q, C3a C4, C5a, and Factor B. This complement pathway activation involves IL-5 and tryptase, more commonly recognized in patients who develop renal failure or fatal cerebral events. Mast cells can also be activated via low-affinity Mas-related G protein-coupled receptor X2 (MRGPRX2), which activates mast cells via non-Fc*ε* receptors. Another possible pathway is through neuropeptides, including corticotropin-releasing hormone, neurotensin (NT), and substance P (SP) via high-affinity receptors [14].

Risk factors of Kounis syndrome include a history of previous allergy, hypertension, smoking, diabetes, and hyperlipidemia. Various causes have been found to trigger Kounis syndrome, and more triggers are being identified. The most common trigger of Kounis syndrome is antibiotics (27.4%), followed by insect bites. Other causes include foods, latex, and dialysate [3]. Medicines that may trigger Kounis syndrome include analgesics, antibiotics, glucocorticoids, nonsteroidal anti-inflammatory drugs, and anticancer drugs.

Regarding Kounis syndrome triggered by antibiotics, cases have been reported on amoxicillin, amikacin, cefuroxime, penicillin, sulbactam/cefoperazone, piperacillin/tazobactam, trimethoprim-sulfamethoxazole, and vancomycin [1]. Clinical studies indicate that allergic patients who are simultaneously exposed to several allergens have more symptoms than monosensitized individuals. The more medicines an atopic patient is exposed to, the easier and quicker anaphylaxis and Kounis syndrome can occur [15]. Our patient was previously allergic to amoxicillin, flucloxacillin, clindamycin, and tetracycline. Nevertheless, the general practitioner had put on flucloxacillin and clindamycin without taking a proper allergic history. After identifying the allergic reaction, the antibiotics were changed to cephalexin, and the patient developed Kounis syndrome. Thus, there is a possibility that this patient was exposed to three possible allergens, which would have easily triggered Kounis syndrome [15].

A case of clindamycin-triggered Kounis syndrome has been reported in a 17-year-old boy who has taken treatment for acute tonsillitis and subsequently developing acute anterolateral myocardial infarction with ST-segment elevation [12]. Our patient was treated with clindamycin and flucloxacillin, which might be the possible triggers of Kounis syndrome, manifested as acute anterolateral myocardial infarction with ST-segment depression in V4–V6 leads. However, it is difficult to determine which caused the symptoms since the patient claimed to be allergic to both antibiotics. There are no reported cases of Kounis syndrome secondary to flucloxacillin.

The diagnosis of Kounis syndrome is based on clinical symptoms and signs and laboratory, electrocardiographic, echocardiographic, and angiographic evidence. A variety of these findings might accompany allergic symptomatology that helps to establish the correct diagnosis. The symptoms include acute chest pain, chest discomfort, dyspnea, faintness, headache, nausea, pruritus, and skin itching. Therefore, entertaining a high index of paramount suspicion is important. Patients with systemic allergic reactions associated with clinical, electrocardiographic, and laboratory findings of acute myocardial ischemia should be suspected of having Kounis syndrome [1]. Newer diagnostic techniques such as cardiac magnetic resonance imaging (MRI) and myocardial scintigraphy can help to confirm the diagnosis. Increased serum tryptase, histamine, cardiac biomarkers, and cardiac troponins are beneficial findings [12]. In this case, the patient developed an ischaemic-type chest pain, and the immediately taken electrocardiogram showed ischaemic changes. He had an elevated troponin I level with symptoms of hypersensitivity, and the diagnosis of Kounis syndrome was made.

Management of Kounis syndrome is a complex task that requires rapid treatment decisions aimed at myocardium revascularisation and treating the concomitant allergic reaction. Currently, guidelines for the treatment of Kounis syndrome are lacking. Most of the evidence on the efficacy of the treatment is based on individual case reports or case series [16]. A recent review conducted by Kounis himself recommends different management approaches for the multiple kinds of variants [2].

The two main aspects of management include cardiological evaluation and treatment of ACS and emergency treatment of acute allergic reactions. As initial steps for managing ACS, nitroglycerine infusion or tablets can be given, and calcium channel blockers such as verapamil or diltiazem can be considered. Intramuscular epinephrine, oxygen therapy, intravenous fluids, glucocorticoid, and H1-blockers can be given to manage the allergic reaction after removing the tentative allergen. Next, continuous monitoring is indicated for the type I variant, and dual antiplatelet therapy and percutaneous coronary intervention can be performed for type II and type III variants. It is also essential to evaluate the ejection fraction and hemodynamic instability. Timely referrals for the CCU or ICU should be done [16]. This patient was managed initially with nitroglycerin sublingual tablet and next with dual antiplatelet therapy. Six doses of subcutaneous enoxaparin and prednisolone to control the allergic reaction were also given. He was discharged with nitrates, dual antiplatelets, and antihypertensives, considering his chronic medical conditions. According to the literature, although metoprolol was given to this patient, it is contraindicated in Kounis syndrome. Beta-blockers can aggravate coronary vasospasms in patients with type II Kounis syndrome [17].

So far, there are only seven cases of Kounis syndrome reported in Sri Lanka. Among them, five cases have depicted ST-elevation myocardial infarctions. One case has reported amoxicillin/clavulanic acid-induced Kounis syndrome with widespread ST elevations [18]. In another case, the patient has presented with acute ST-elevation myocardial infarction associated with peripheral blood eosinophilia without any obvious trigger of hypersensitivity [19]. Two other cases of Kounis syndrome have been reported following snake bites, one following a hump-nosed pit viper bite [17] and another a cobra bite [20]. Another case has been reported following a hornet sting causing widespread T inversions in the electrocardiogram [21]. In two cases, the patients have succumbed following Kounis syndrome, one following ceftazidime injection where postmortem found myocardial necrosis with elevated tryptase level suspecting the diagnosis of Kounis syndrome [22]. The other fatal case of Kounis syndrome was an anterior ST-elevation myocardial infarction following anaphylaxis after ingestion of prawns [23]. Therefore, we report the second case of Kounis syndrome with non-ST-elevation of myocardial infarction and the first one with ST-segment depression in Sri Lanka.

## 4. Conclusions

This case emphasizes that physicians should be aware of allergic angina syndrome, also known as Kounis syndrome, when a patient simultaneously develops symptoms of ACS and hypersensitivity features. In such cases, other supportive investigations such as electrocardiograms and cardiac biomarkers should be conducted to support the diagnosis, and the management should be initiated promptly. Peripheral blood eosinophilia also supports the diagnosis of Kounis syndrome even in the absence of other features of hypersensitivity. Kounis syndrome is a life-threatening clinical entity regardless of the triggering allergen. Since the management of Kounis syndrome is more complex than solitary ACS, the recognized bodies need to make an evidence-based clinical guideline to manage the condition effectively. At the same time, pharmacovigilance is essential when prescribing medicines, especially when a patient has a significant allergic history.
